# Proof of concept: Screening for REM sleep behaviour disorder with a minimal set of sensors

**DOI:** 10.1016/j.clinph.2021.01.009

**Published:** 2021-04

**Authors:** Navin Cooray, Fernando Andreotti, Christine Lo, Mkael Symmonds, Michele T.M. Hu, Maarten De Vos

**Affiliations:** a University of Oxford, Institute of Biomedical Engineering, Dept. Engineering Sciences, Oxford, UK; b Oxford Parkinson's Disease Centre (OPDC), University of Oxford, UK; c Nuffield Department of Clinical Neurosciences, University of Oxford, UK; d Department of Clinical Neurophysiology, John Radcliffe Hospital, Oxford University Hospitals Foundation Trust, UK

**Keywords:** Automated sleep staging, Electrocardiogram, Electrooculogram, Electromyography, Parkinson’s disease, Polysomnography, REM sleep behaviour disorder, RBD, Sleep diagnostic tool

## Abstract

**Objective:**

Rapid-Eye-Movement (REM) sleep behaviour disorder (RBD) is an early predictor of Parkinson’s disease, dementia with Lewy bodies, and multiple system atrophy. This study investigated the use of a minimal set of sensors to achieve effective screening for RBD in the population, integrating automated sleep staging (three state) followed by RBD detection without the need for cumbersome electroencephalogram (EEG) sensors.

**Methods:**

Polysomnography signals from 50 participants with RBD and 50 age-matched healthy controls were used to evaluate this study. Three stage sleep classification was achieved using a random forest classifier and features derived from a combination of cost-effective and easy to use sensors, namely electrocardiogram (ECG), electrooculogram (EOG), and electromyogram (EMG) channels. Subsequently, RBD detection was achieved using established and new metrics derived from ECG and EMG channels.

**Results:**

The EOG and EMG combination provided the optimal minimalist fully-automated performance, achieving 0.57 ± 0.19 kappa (3 stage) for sleep staging and an RBD detection accuracy of 0.90 ± 0.11, (sensitivity and specificity of 0.88 ± 0.13 and 0.92 ± 0.098, respectively). A single ECG sensor achieved three state sleep staging with 0.28 ± 0.06 kappa and RBD detection accuracy of 0.62 ± 0.10.

**Conclusions:**

This study demonstrates the feasibility of using signals from a single EOG and EMG sensor to detect RBD using fully-automated techniques.

**Significance:**

This study proposes a cost-effective, practical, and simple RBD identification support tool using only two sensors (EMG and EOG); ideal for screening purposes.

## Highlights

• We demonstrate the feasibility of a fully automated REM sleep behaviour (RBD) screening tool using minimal sensors.
• REM sleep is detected accurately and reliably in individuals with RBD, without EEG sensors.
• Automated sleep staging was able to classify REM sleep with sufficient accuracy to allow for the accurate detection of RBD.

## 1 Introduction

In the search for an early predictor for the onset of Parkinson’s disease (PD), few are as promising as Rapid-Eye Movement (REM) Sleep behaviour disorder (RBD). Recent literature estimates a rate of 73.5% conversion of RBD to an overt neurodegenerative syndrome over a 12-year follow-up period, with a 6% risk of conversion per year (Postuma et al., 2019). To further explore the mechanisms behind RBD phenoconversion and to develop neuroprotective therapies, larger cohorts of individuals with RBD must be identified. The greatest hurdle to designing such studies is the slow and arduous RBD diagnostic process, involving polysomnography (PSG) recordings within sleep clinics, which are often under-staffed and oversubscribed (severely limiting availability). PSG recordings are expensive, time consuming to setup, potentially uncomfortable, and rely on labour intensive visual inspection of PSG data. Previous studies have demonstrated that automated sleep staging and RBD detection is possible from a limited PSG, consisting of electroencephalogram (EEG), electrooculogram (EOG), and electromyogram (EMG) sensors (Cooray et al., 2018, Cooray et al., 2019). This work sought to further minimise this limited framework by excluding EEG sensors and exploring only non-EEG sensors to determine the effective, yet minimal, set of sensors necessary for fully automated RBD detection.

One such non-EEG alternative includes electrocardiogram (ECG) signals, which are ubiquitous and easy to apply. Furthermore, literature provides evidence to suggest that ECG signals are valuable in automated sleep staging and could even be utilised to identify individuals with RBD. Sleep research is uncovering an interplay between the central nervous system and the autonomic nervous system (ANS) that is regulated throughout sleep and its various stages (de Zambotti et al., 2018). The ANS is responsible for regulating the majority of the body’s internal systems, such as blood pressure, breathing, body temperature, digestion, micturition, and myocardial function. Heart rate and heart rate variability (HRV) derived from ECG sensors are commonly used to evaluate components of ANS functionality (Andreotti et al., 2018). Typically, HRV is derived from the variation in the interval between QRS complexes found in the ECG signal (measuring the variation in heartbeat intervals). Manually or automatically derived QRS complexes have been used to demonstrate automated three-state sleep staging (wake (W), non-REM (NREM), and REM) (Redmond and Mcnicholas, 2007, Mendez et al., 2010, Xiao et al., 2013, Ebrahimi et al., 2015, Fonseca et al., 2015, Malik et al., 2018, Yücelbaş et al., 2018).

Additionally, literature has identified cardiac autonomic dysfunction as a characteristic of RBD (Postuma et al., 2010, Sorensen et al., 2013, Bugalho et al., 2018, Knudsen et al., 2018). Postuma et al. (2010) demonstrated through the R-peak interval (RR) standard deviation and low frequency components, clear autonomic dysfunction in RBD participants compared to healthy controls (during wakefulness) (Postuma et al., 2010). Similarly, Sorensen et al. (2013) illustrated attenuated sympathetic activity in HRV (Sorensen et al., 2013). Furthermore, Bugalho et al. (2018) described a reduction in the parasympathetic modulation of HRV in relation to sleep stage transitions (Bugalho et al., 2018). Also consistent with these findings, a multimodal imaging study conducted by Knudsen et al. (2018) found evidence for profound cardiac sympathetic denervation using ^123^I-metaiodobenzylguanidine scintigraphy (Knudsen et al., 2018). Therefore ECG along with EOG and EMG sensors provide an opportunity to further economise sleep staging and RBD detection by excluding the need for cumbersome and expensive EEG sensors.

The potential for minimalist automated sleep staging has been demonstrated in single sensor studies that used EOG or EMG sensors to replicate the performance of EEG electrodes (Virkkala et al., 2007, Virkkala et al., 2008, Yetton et al., 2016). With respect to RBD detection, our previous work utilised established objective techniques to evaluate REM sleep without atonia (RWSA) and demonstrated how they could be improved using a combination of EMG metrics that incorporate sleep architecture (Cooray et al., 2018, Cooray et al., 2019). This paper investigated the inclusion of ECG based metrics that exploit changes in ANS, as a substitute or enhancement for RBD detection. Additionally, this study compared the accuracy of using a simplified combination of ECG, EOG, and EMG sensors to that of a conventional PSG in screening for RBD.

## 2 Data

PSG recordings used in this study were collected from several sources and included participants diagnosed with RBD and age-matched healthy controls (HCs), detailed in Table 1. The Montreal Archive of Sleep Studies (MASS) cohort one (O’Reilly et al., 2014) provided 53 HC individuals, where three were excluded due to poor QRS detection quality (specifically, participants labelled SS01, SS10, and SS18). Clinically diagnosed participants with RBD were collated from the Physionet Cyclic Alternating Pattern (CAP) sleep database (22 RBD participants) (Goldberger et al., 2000, Terzano et al., 2001) and a private database of RBD participants from the John Radcliffe (JR) hospital (35 participants), Nuffield Department of Clinical Neurosciences at the University of Oxford. While the JR dataset included two nights of full PSG recordings for each participant, this study used the second night, where available (only two participants had a single night recording). Eight recordings from the CAP database were excluded because one was a duplicate recording (RBD11) and seven had secondary RBD, including five diagnosed with PD (RBD1, RBD2, RBD3, RBD7, and RBD9), one with multiple systems atrophy (RBD13), and one with Lewy body disease (RBD5). Consequently, this combined RBD cohort provided a total of 50 RBD participants, of which two were taking Clonazepam to treat their condition, while ten participants were taking anti-depressants (Citalopram, Venlafaxine, Sertraline, Duloxetine, and Lorazepam). All RBD participants had an apnoea-hypopnea index (AHI) of less than 7.1, which was evaluated as being mild and unremarkable, except for one participant (RBD1) from the CAP sleep database with a severe index of 30/h (already excluded for secondary RBD). Five RBD participants with concurrent RBD and obstructive sleep apnoea (OSA) wore a continuous positive airway pressure ventilator at the time of their PSG recordings, which would benefit their AHI score. This study complied with the requirements of the Department of Health Research Governance Framework for Health and Social Care 2005 and was approved by the Oxford University hospitals NHS Trust (HH/RA/PID 11957). These two RBD datasets were combined to balance the HC recordings provided by the MASS database. Furthermore, this combination provided an opportunity to evaluate how generalizable this study is over differing datasets annotated at different institutions. Once more by using openly available datasets (MASS and CAP), this study can be reproduced in combination with the toolbox provided at https://github.com/navsnav/Minimal-RBD-Sleep-Detection.

**Table 1:** Datasets used in the study. Recordings from individuals with REM sleep behaviour disorder (RBD) were collected from the John Radcliffe (JR) hospital and the Cyclic Alternating Pattern (CAP) database, which were combined into a single cohort (CAP/JR). Note the male predominance within the RBD dataset. Healthy control (HC) participants were sourced from the Montreal Sleep Studies (MASS) database. These datasets are analysed separately for sleep stage classification and combined for RBD detection using electrocardiogram and electromyogram metrics.

Database	Cohort	Age	#Subjects	#Female	#Male
CAP	RBD	70.2 ± 5.3	14	2	12
JR	RBD	64.3 ± 8.0	36	2	34
Combined (CAP/JR)	**RBD**	**68.0 ± 7.8**	**50**	**4**	**46**
MASS	**HC**	**63.7 ± 5.2**	**50**	**17**	**33**

Annotations of the PSG recordings were completed by experts using either the Rechtschaffen and Kales (R&K) (Rechtschaffen and Kales, 1968) or AASM guidelines (Berry et al., 2012). For the purposes of this study, each set of rules were converted to three stages (REM, NREM, and W), where NREM was defined by S1, S2, S3, S4, N1, N2, or N3. To explore the most economical combination of sensors, the following signals were analysed:

• 1 ECG (2 electrodes - Einthoven derivation)
• 1 EOG (2 electrodes – bipolar signal)
• 1 EMG (2 electrodes – submentalis)

Numerous studies have used limited sensors to assess automated sleep staging, but in this study we applied automated sleep staging to the PSGs of healthy controls and RBD participants, followed by automated RBD identification.

## 3 Method

### 3.1 Pre-processing

To reduce the impact of noise and artefacts, PSG signals were pre-processed. Firstly, all ECG, EOG, and EMG signals were resampled at 200 Hz. The EOG signal was pre-processed with a 500th order band pass finite impulse response (FIR) filter with a cut-off frequency of 0.3 Hz and 40 Hz. The EMG signal was filtered with a 500th order notch filter at 50 Hz and 60 Hz (because the recordings were sourced from either Europe or Canada), in addition to a 500th order band pass FIR filter between 10 Hz and 100 Hz. The ECG signal was filtered by a 10th order Butterworth band pass filter between 5 Hz and 45 Hz.

### 3.2 Feature extraction

Feature extraction for EOG and EMG signals were as described in our previous study (Cooray et al., 2019), and based on established automated sleep staging literature (Güneş et al., 2010, Koley and Dey, 2012, Liang et al., 2012, Lajnef et al., 2015, Yetton et al., 2016). Literature that described ECG features for automated sleep staging provided motivation for this study (Redmond and Mcnicholas, 2007, Mendez et al., 2010, Xiao et al., 2013, Ebrahimi et al., 2015, Fonseca et al., 2015, Yoon et al., 2017, Malik et al., 2018, Yücelbaş et al., 2018) and are summarised in Table 2. For this study pre-processed ECG signals were segmented into five-minute epochs, often done for HRV analysis (Ebrahimi et al., 2015). Features were then derived after Pan-Tompkins QRS detection (Pan and Tompkins, 1985) from each 30-s segment. Features were then averaged across a moving 150-s sliding window, as described in other similar studies (Redmond and Mcnicholas, 2007, Xiao et al., 2013, Fonseca et al., 2015).

**Table 2:** Features extracted for automated sleep staging, specifically detailing those from an electrocardiogram (ECG) signal. All electrooculogram (EOG) and electromyogram (EMG) features were detailed in a previous study (Cooray et al., 2019).

Channel	Category	Features	Reference
ECG	Heart beat regularity	Irregular Index (IrrIndex), Origin Count, and evidence of compensatory pauses (PACEv)	(Sarkar et al., 2008)
ECG	R-peak intervals (RR)	Mean and median RR interval, standard deviation of the RR interval (SDNN), square root of mean of squares of difference between adjacent RR intervals (SDNN), square root of mean of squares of difference between adjacent RR intervals (RMSSD), standard deviation of differences between adjacent RR intervals (SDSD), number of pairs of adjacent RR intervals differing by more than 50 ms (NN50), and percentage NN50 (pNN50, NN50 divided by the total number of RR intervals).	(Andreotti et al., 2018)
ECG	Time series	Zero crossing interval (ZCI), mean zero crossing interval (mZCI), variation of amplitude (AmpVarsqi), standard deviation of amplitude (Ampstdsqi), and mean amplitude (AmpMean).	(Andreotti et al., 2018)
ECG	Peak frequency and power	Low frequency peak (LFpeak), high frequency peak (HFpeak), total power, low frequency power (LFpower), high frequency power (HFpower), low frequency power percentage (nLF), high frequency power percentage (nHF), and low and high frequency ratio (LFHF). All features were also calculated with a normalised value.	(Andreotti et al., 2018)
ECG	Non-linear	Standard deviation of Poincare plot (PoincareSD1 and PoincareSD2), sample entropy, approximate Entropy, recurrence rate, determinism (DET), Shannon entropy (ENTR), diagonal line length (L), Teager-Kaiser energy operator (TKEO), detrended fluctuation analysis exponent (DAFa2), Lempel Ziv complexity (LZ), mutual information (PD and BD), auto-correlation (BDa and PDa)	(Andreotti et al., 2018)
ECG	Heart beat	Tachycardia (Tachy, > 100 beats per minute (bpm) in adults) and bradycardia (Brady, < 60 bpm in adults).	(Andreotti et al., 2018)
NA	Elapsed time	Hours recorded from start (HoursRec) and time in hours from the end of the recording (HoursRecEnd).	(this work)

### 3.3 Automated sleep stage classification

Our previous work demonstrated that accurate automated sleep staging (especially REM detection) yielded a high performance in RBD detection (Cooray et al., 2019). This study followed the same methodology and once again the Random Forest (RF) algorithm (Breiman, 2001) was used for automated sleep staging. The classifier was trained to classify 30-s epochs into one of three sleep stages (REM, NREM, and W) using a combination of 25 EOG features, 17 EMG features, and 75 ECG features, as described in Table 2 (the number of trees was set to 500, $m_{\mathrm{try}}=\sqrt{M}$ (rounded down) randomly selected features, where M is the total number of features (detailed in Table 3)). For each of the three sleep stages the classifiers were evaluated using macro-averaged sleep stage accuracy, sensitivity, specificity, and Cohen-Kappa score (three stage) by using 10-subject-fold cross-validation with an even split between healthy and RBD participants. Additionally, multi-stage classification was assessed by Cohen’s Kappa (Cohen, 1960), a metric often used in sleep staging to evaluate the agreement between manual and automated annotations. Performance was compared to results obtained using EEG, EOG, and EMG features described in Cooray et al. (2019).

**Table 3:** Combination of polysomnography sensors used and analysed for automated sleep staging (three states), which included electrocardiogram (ECG), electromyogram (EMG), and electrooculogram (EOG) signals. The Z3 combination represented our previous study and was used as a comparison to determine the merits of electroencephalogram (EEG) features (Cooray et al., 2019).

Sleep Staging Signals	#Features	#Sensors	ID
ECG	75	1	A1
EOG	25	1	B1
EMG	17	1	C1
ECG + EOG	98	2	A2
ECG + EMG	90	2	B2
EOG + EMG	40	2	C2
ECG + EOG + EMG	113	3	A3
EEG + EOG + EMG	128	3	Z3

### 3.4 RBD detection

A diagnosis of RBD mandates the visual confirmation of RSWA following the identification of REM sleep (Sateia, 2014). In our previous work we demonstrated that established features, which quantify RSWA in combination with sleep architecture, provided RBD detection performances approaching that of the gold standard (manual clinical diagnosis) by using RF classifiers (Cooray et al., 2018, Cooray et al., 2019). This study emulated previous results and compared them to RBD detection using additional ECG based metrics in isolation and combination with EMG based metrics through RF classification.

RF classifiers were used to achieve RBD detection, as per the combinations detailed in Table 4. Classifiers were trained using 500 trees; $m_{\mathrm{try}}=\sqrt{M}$ (where M is the number of features detailed in Table 4), and evaluated using a 10-subject-fold cross-validation scheme. In addition to the features detailed in our previous study, we proposed new ECG features that capture changes in HRV between sleep stages (see Table 5).

**Table 4:** These are the various polysomnography sensor combinations analysed for REM sleep behaviour disorder (RBD) detection in coordination with automated sleep staging. Metrics derived from electrocardiogram (ECG) sensors are detailed in Table 5, while electromyogram (EMG) metrics were described in a previous study (Cooray et al., 2019).

RBD Detection Signals	#Features	#Sensors	ID
ECG	11	1	D1
EMG	7	1	E1
ECG + EMG	18	2	D2

**Table 5:** Electrocardiogram (ECG) metrics for REM sleep behaviour disorder (RBD) detection, which aimed to capture changes in heart rate variability during sleep.

Channel	Category	Description	Reference
ECG	Time	The mean RR interval standard deviation during all REM epochs (RR_REM_Std),	(Andreotti et al., 2018)
ECG	Frequency	The mean RR interval low frequency peak during NREM epochs (LFpeak_NREM), mean RR interval low frequency peak during REM epochs (LFPeak_REM), mean RR interval high frequency peak during NREM epochs (HFpeak_NREM), and the RR interval high frequency peak during REM epochs (HFPeak_REM).	(Andreotti et al., 2018)
ECG	Non-linear	The mean sample entropy during REM (SampEn_REM).	(Andreotti et al., 2018)
ECG	Heart beat regularity	The mean irregular index (IrrIndex_NREM), the mean irregular index calculated for each REM epoch (IrrIndex_REM), the mean origin count for all NREM epochs (OriginCount_NREM), and mean origin count for all REM epochs (OriginCount_REM)	(Sarkar et al., 2008)
ECG	RR index	The mean RR interval ratio between REM and NREM epochs (RR_Index).	This work
ECG	LFHF ratio	The mean RR interval low frequency to high frequency ratio between NREM and REM epochs (LFHF_Index).	This work
NA	NREM ratio	Ratio of NREM sleep compared to REM sleep (Ratio_NREM).	This work

A summary of all ECG based metrics that incorporate sleep architecture and HRV, with the aim of identifying RBD, are detailed in Table 5. Specifically, to quantify HRV, the mean sample entropy for each REM epoch was calculated for every participant. Similarly the standard deviation of the RR intervals for each REM epoch was averaged for each participant. Often low frequency and high frequency components of RR intervals are used to describe sympathetic and parasympathetic activity in the ANS. The mean peak low frequency and high frequency was therefore calculated for all participants. To capture changes in heart rate variation between sleep stages, the ratio between REM and NREM values of the mean RR interval were calculated. The low frequency and high frequency ratio is a popular feature used in HRV and the ratio between NREM and REM epochs was calculated for each participant as an RBD metric. Literature has also suggested that PD is significantly comorbid with atrial fibrillation (AF) (Hong et al., 2019). Consequently the metrics termed irregular index and origin count (Sarkar et al., 2008, Oster and Clifford, 2015) were used to quantify irregularly irregular heartbeats during NREM and REM sleep. These ECG based metrics were used to automatically identify RBD participants and were compared to metrics from our previous studies (Cooray et al., 2018, Cooray et al., 2019).

Furthermore the RBD detection performance of these metrics were evaluated using manually and automatically annotated sleep staging using the optimal combination of minimal sensors (see Table 3), in order to validate their use in an end-to-end RBD screening support tool. The impact of automated sleep staging on the calculation of these metrics was also carefully analysed.

Bland and Altman plots (B&A) were introduced to describe the agreement between two quantitative measures as an improvement to simple correlation factors (Bland and Altman, 1986, Bland and Altman, 1995, Bland and Altman, 1999). This study used B&A plots to evaluate the agreement between RBD metrics derived from manual and automatic sleep staging. The B&A plot measures agreement by plotting the mean against the difference of both quantitative methods (Bland and Altman, 1986), while constructing the limits of agreement (where Bland and Altman et al., recommend that 95% of all data points fall within two standard deviations of the mean difference). Furthermore, B&A plots detail the bias between the mean differences of both quantitative methods and their significance. For this study B&A plots were used to establish a level of agreement between RBD metrics, derived using manually and automatically annotated sleep stages within a certain agreement interval. The decision on whether these intervals are acceptable must be decided by clinical evaluation and in our case through the performance of automated RBD detection with metrics derived from automated sleep staging.

## 4 Results

Results are presented in three sections: 1) automated sleep stage classification, 2) RBD detection using automatically annotated sleep stages, and 3) Bland and Altman plots for RBD metrics using automatically/manually annotated sleep stages.

### 4.1 Automated sleep stage classification

**Fig. 1:**
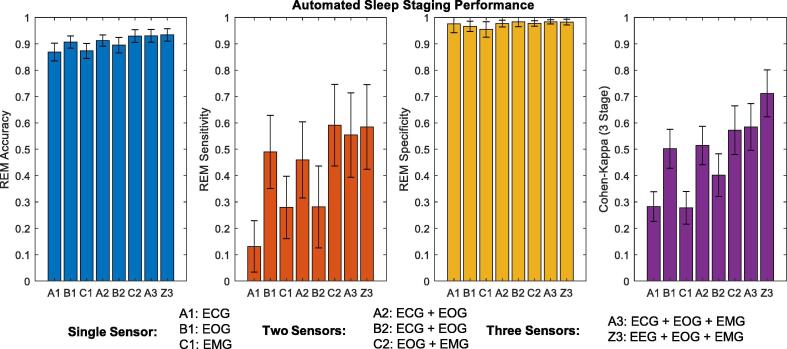
The performance of automated sleep staging for various polysomnography sensor combinations, detailing rapid-eye movement (REM) accuracy, REM sensitivity, REM specificity, and the 3-stage Cohen Kappa score. The best sensitivity and F1-score, for a single sensor combination (A1, B1, and C1), was given by the electrooculogram (EOG) sensor (B1). For two sensors, (A2, B2, C2), the best performance was given by the EOG and electromyogram (EMG) combination(C2). This exceeded the performance of a single EOG sensor (B1), which is illustrated by the increase in sensitivity and near equal specificity between C1 and B1. The inclusion of the electrocardiogram (ECG) does little to improve the performance of automated sleep staging (A1, A2, B2, and Z3) compared to combinations without the ECG sensor. The Z3 combination provides a comparison to a combination that includes an electroencephalogram (EEG) sensor.

The best performance of automated sleep staging was provided by all three signals (EOG, ECG, and EMG) attaining an agreement kappa score of 0.58 (0.70 and 0.48 for the individual HC and RBD cohort, respectively). These results are also comparable to our previous study that included EEG features (Cooray et al., 2019), shown in Fig. 1 as combination Z3 (EEG, EOG, and EMG). Once again it is clear that the automated sleep staging performs considerably better on HC participants than RBD participants, echoing the results from our previous study (Cooray et al., 2019). The best performing combination of two sensors proved to be the EOG and EMG sensors (C2), followed by the EOG and ECG combination (A2) achieving 0.57 and 0.51 agreement kappa scores, respectively. The combination of ECG and EMG features resulted in an agreement kappa score of 0.41. The best single sensor was the EOG; features (see Fig. 1, B1) achieving a three-stage score of 0.50, considered moderate agreement (Landis and Koch, 1977). The single ECG and EMG sensor (A1 and C1, respectively) had limited success, never exceeding fair agreement (0.30 and 0.28, respectively). The performance of the optimum sensor combination (C2) for automated sleep staging is detailed in Table 6.

**Table 6:** Performance of automatic sleep stage classification using combined electrooculogram (EOG) and electromyogram (EMG) features (sensor combination C2). The performance of using both EOG and EMG channels was substantially better than using a single electrocardiogram (ECG), EMG, or EOG channel. Importantly for REM sleep behaviour disorder (RBD) detection, REM specificity and precision was relatively high.

	Wake	Non-REM	REM
Accuracy	0.85 ± 0.10	0.81 ± 0.098	0.93 ± 0.047
Sensitivity	0.64 ± 0.21	0.90 ± 0.12	0.60 ± 0.31
Specificity	0.92 ± 0.11	0.66 ± 0.18	0.98 ± 0.022
Precision	0.73 ± 0.20	0.83 ± 0.096	0.76 ± 0.22
F1	0.64 ± 0.17	0.85 ± 0.097	0.62 ± 0.27

**Fig. 2:**
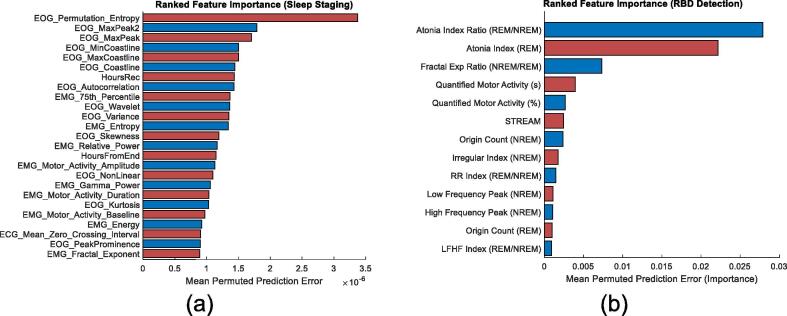
The order of feature importance for (a) automated rapid-eye movement (REM) classification (sleep staging) using electrocardiogram (ECG), electrooculogram (EOG), and electromyogram (EMG) features (top 25) and (b) REM sleep behaviour (RBD) detection (top 13). From (a) the EOG features appear most important for REM classification, specifically permutation entropy, max peak, and coastline features. Followed by elapsed recording time and EMG features, such as the 75th percentile, entropy, relative power, and motor activity. These additional features supplement the EOG features to provide a boost in REM classification performance, shown in Fig. 1 (see sensitivity of B1 and C2). From (b) the feature importance for RBD detection clearly illustrated that EMG metrics outperform ECG metrics. Of the ECG metrics, irregular evidence (origin count and irregular index) during non-REM (NREM) appeared the most effective for RBD detection. These are followed by frequency-based ECG metrics, such as low frequency peak, high frequency peak, and the low frequency to high frequency (LFHF) index.

For REM classification, the ranked importance of features is detailed in Fig. 2 (a), when using a combined set of EOG, ECG, and EMG features. In Fig. 2 (a) EOG features dominate the list, while EMG and ECG features make an appearance in the top 25.

### 4.2 RBD detection with ECG and EMG metrics

**Fig. 3:**
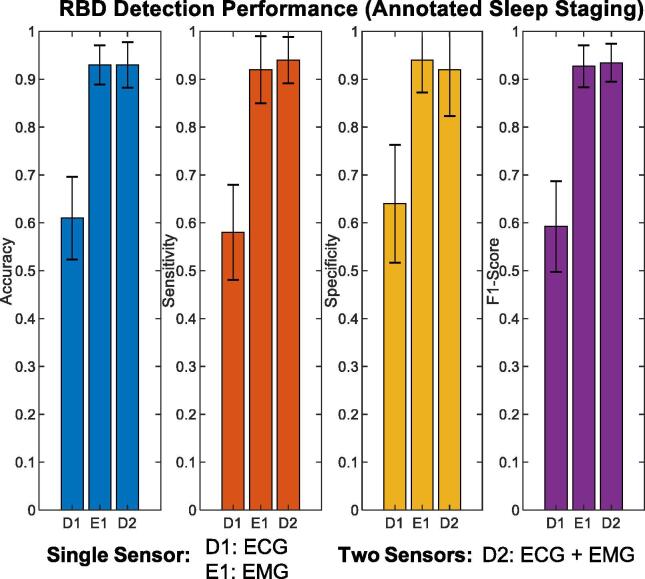
Performance of rapid-eye movement sleep behaviour disorder (RBD) detection using electrocardiogram (ECG-D1), electromyogram (EMG-E1), and both ECG and EMG signals (D2) with manually annotated sleep stages. With manual annotations, ECG metrics (D1) appeared effective at RBD identification, but are clearly outperformed by EMG metrics (E1). Combining these sensors (D2) only improved performance marginally with respect to the F1 score.

The accuracy of RBD detection using ECG and EMG metrics is depicted in Fig. 3. ECG metrics distinguished RBD participants with an accuracy, sensitivity, and specificity of 0.62 ± 0.20, 0.56 ± 0.23, and 0.68 ± 0.24, respectively**.** The combination of EMG metrics and sleep architecture (detailed in our previous study) achieved an accuracy, sensitivity, and specificity of 0.93 ± 0.09, 0.92 ± 0.13, and 0.94 ± 0.092**,** respectively. Combining EMG and ECG metrics for RBD detection provided a similar accuracy, sensitivity, and specificity of 0.93 ± 0.10, 0.94 ± 0.092, and 0.92 ± 0.13, respectively.

The ranked importance of ECG and EMG metrics for RBD detection are depicted in Fig. 2 (b), where EMG metrics, specifically the atonia index ratio (between NREM and REM), atonia index (REM), and the fractal exponent ratio (between NREM and REM), proved to be the most important.

**Fig. 4:**
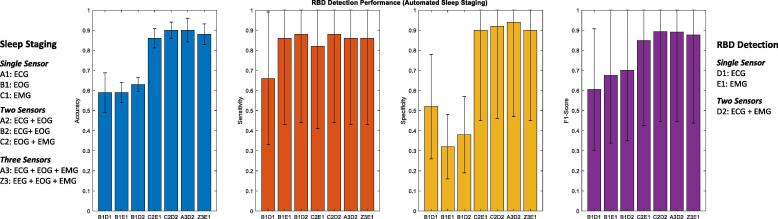
Rapid-eye movement sleep behaviour disorder (RBD) detection performance using automated sleep staging. For this figure B1, C2, and A3 represent automated sleep staging using electrooculogram (EOG), both EOG and electromyogram (EMG), and electrocardiogram (ECG), EOG, and EMG features, respectively. While D1, E1, and D2 represent metrics derived from EMG, ECG, and both EMG and ECG sensors for RBD detection, used in combination with automated sleep staging. The Z3D1 combination represents the results from our previous study that included electroencephalogram (EEG), EOG, and EMG features for automated sleep staging and EMG metrics for RBD detection. From this figure we can observe how non-EEG sensors (C2E1, C2D2, and A3D2) can achieve fully automated RBD detection similar to EEG sensors (Z3D1).

Fig. 4 details the best RBD detection performance from automated sleep staging classifiers using a combination of EOG, ECG, and EMG features. The best performance (F1 score) was associated with the C2D2 combination (three sensors - EOG and EMG for sleep staging followed by EMG and ECG for RBD detection), followed by C2E1 combination (two sensors - EOG and EMG for sleep staging followed by EMG for RBD detection) with an accuracy, sensitivity, specificity, and F1 score of 0.90 ± 0.11, 0.88 ± 0.13, 0.92 ± 0.098, and 0.90 ± 0.12. These results are similar and only marginally less than when using a cumbersome EEG combination (Cooray et al., 2019), depicted by Z3 (Fig. 4).

As described later in our Discussion, the EMG and EOG sensor was deemed the most ergonomic for fully-automated RBD detection, where the overall performance is detailed in Table 7 (achieving a mean accuracy, sensitivity, and specificity of 0.90, 0.88, and 0.92, respectively).

**Table 7:** REM sleep behaviour disorder (RBD) detection results using automatically annotated sleep stages. Automated sleep staging was achieved using electrooculogram and electromyogram (EMG) features (C2 combination), where Fig. 1 detailed this sensor combination as the most parsimonious. RBD detection results are compared using the atonia index (an established metric used in literature) and random forest (RF) models trained using metrics from sensor combinations E1, D1, and D2 (detailed in Table 4). While RBD detection using an RF model trained on EMG and electrocardiogram (ECG) metrics (D2 combination) provided the best F1-score, this was only marginally better than the RF model trained on only EMG metrics (E1 combination). These results indicate that the C2E1 as the most parsimonious for fully-automated RBD detection using features and metrics derivied from EOG and EMG sensors.

RBD Detection	RBD Detection using Automatic Sleep Staging (C2)			
	Accuracy	Sensitivity	Specificity	F1
Atonia Index	0.83 ± 0.12	0.68 ± 0.22	0.98 ± 0.06	0.78 ± 0.18
RF (D1:ECG)	0.65 ± 0.22	0.70 ± 0.24	0.60 ± 0.24	0.66 ± 0.21
RF (E1:EMG)	0.90 ± 0.11	0.88 ± 0.13	0.92 ± 0.098	0.90 ± 0.12
RF (D2:EMG+ECG)	0.92 ± 0.098	0.88 ± 0.13	0.96 ± 0.08	0.91 ± 0.11

### 4.3 RBD metrics from automated and annotated sleep staging (Bland-Altman)

**Fig. 5:**
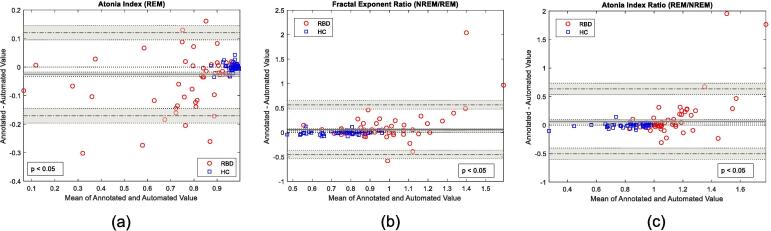
Bland and Altman plots of important rapid-eye movement (REM) sleep behaviour disorder (RBD) metrics comparing scores derived from manually and automatically annotated sleep stages. These included the (a) atonia index during REM sleep, (b) atonia index ratio between REM and Non-REM (NREM), and the (c) fractal exponent ratio between REM and NREM. In all cases we can observe that metrics are within the limits of agreement (Bland and Altman, 1986). The p-value (p) details the Kolmogorov-Smirnov test to determine if the difference between metrics calculated from manual and automatic sleep staging are from a normal distribution (rejected when < 0.05). For metrics shown above, the line of equality (the dotted line at zero) does not fall within the limits of confidence around the mean difference (shaded grey area). Therefore when using automated sleep staging the calculated metrics for RBD detection slightly shifts towards values associated with RBD or healthy control (HC) participants. This is unsurprising given misclassification from automated sleep staging will have an impact on the calculation of these metrics.

The changes in RBD metrics calculated from automated sleep staging from a EOG and EMG sensor (compared to manual annotation) was evaluated by the B&A plots given in Fig. 5. A selection of important RBD metrics (provided by Fig. 2 (b)) appears to provide agreeable results, where the limits of agreement are represented by horizontal dotted lines. The mean difference bias was defined by the solid black line in Fig. 5, where the top three RBD metrics all appear to have a slight bias when using automated sleep staging (shaded area around mean difference includes zero).

## 5 Discussion

The aim of this study was to investigate a minimalistic approach towards identifying RBD through a fully automated pipeline, using a rudimentary dataset (containing two cohorts of healthy controls and RBD participants) as a proof of concept. This study excluded the use of cumbersome-to-apply EEG sensors and instead focused on a combination of ECG, EOG, and EMG sensors to achieve automated three-stage sleep classification and RBD identification. The use of EOG and EMG sensors for automated sleep staging proved the most effective, achieving REM detection that was comparable with the literature and near human expert annotation (Fig. 1). This is not surprising, given that visual staging (especially REM) relies heavily on EOG and EMG signals. In this study ECG features appear ineffective in automated sleep staging for a mixed cohort of HC and RBD participants (compared to those derived from EOG and EMG signals). This study also validated the use of ECG based RBD metrics to identify RBD using an RF classifier (Fig. 3). Once more the performance of RBD detection remained high when using automated sleep staging from EOG and EMG features (Fig. 4 and Table 7). This can be attributed to the high positive predictive value and specificity of REM provided by the automated sleep staging that facilitated congruent RBD metrics (Fig. 5).

As has been established in literature, the inter-rater variability of manual sleep staging is high and is exacerbated further by sleep disorders that provide additional variation in sleep characteristics (Danker-Hopfe et al., 2004, Danker-Hopfe et al., 2009). This was also demonstrated in our previous study, where automated sleep staging performed better on HCs than RBD participants (Cooray et al., 2019) and was again observed in this study (for all combinations of sensors). It was particularly difficult for sleep staging to be performed using a single ECG sensor in RBD participants.

The automated three-stage classification performance of ECG features (see Fig. 1) on a combined dataset (kappa of 0.30), reflected the lower range of results achieved by similar studies (kappa between 0.35 and 0.73) (Redmond and Mcnicholas, 2007, Xiao et al., 2013, Fonseca et al., 2015, Yücelbaş et al., 2018). However these studies utilised smaller datasets focusing on relatively younger HCs (participants of 28–48 people with a mean age that varies from 41 to 43). Yücelbaş et al. (2018) achieved the highest kappa score of 0.74, but even this technique applied to another dataset achieved a kappa of 0.43 and for participants with OSA scored between 0.52 and 0.57, indicating a high degree of variability (Yücelbaş et al., 2018). Xiao et al. (2013) had the added benefit of using manually annotated ECG segments that were considered appropriate, achieving a kappa score of 0.47 (Xiao et al., 2013).

Interestingly, while this study achieves a kappa score of 0.30 (using only ECG features) on a mixed cohort of 100 participants, the RBD cohort severely underperforms with respect to REM detection. This was due to the misclassification of REM states as NREM and W, potentially caused by changes to HRV regulation and movement artefacts, which might be exacerbated by symptoms of RBD. The feature importance for REM detection, detailed in Fig. 2 (a), ranked elapsed-time as more indicative than ECG features. Elapsed time is the progression of time since the beginning of the first annotated epoch from the PSG recording. This feature might benefit from the sleep technician arbitrarily starting to annotate sleep stages from a point they deem appropriate, around the time of lights-off. While this attribute might not be directly applicable to a fully automated take-home screening device, an equivalent starting point for a wearable device could be self-indicated lights off (often done to ensure synchronisation between recording devices). The highest ranked ECG features included RR intervals and zero crossing intervals (ZCI), which are illustrative of HRV but ultimately these features do not contribute towards high REM detection sensitivity.

Alternatively, EOG features provided a boost to automated sleep staging performance (kappa score of 0.50 and mean REM F1 score of 0.52), as shown in Fig. 1. These results are comparable to EOG sleep staging literature that achieved four-state kappa scores of 0.59 (Virkkala et al., 2008) and REM F1 scores of 0.64–0**.**80 (Agarwal et al., 2005, Virkkala et al., 2008, Yetton et al., 2016). It is clearly evident that for automated sleep staging, EOG features outperform ECG features. Combining EMG and EOG features, further improved automated sleep staging with a kappa of 0.57 and mean REM F1 score of 0.62. The use of all three sensors (EOG, EMG, and ECG) provided an almost negligible improvement (kappa score of 0.58 and mean REM F1 score of 0.60) indicating no additional merit for an additional ECG sensor, which would add cost and hassle to a potential screening/diagnostic support tool. The inclusion of EEG features (Z3 combination, see Fig. 1), as per our previous study (Cooray et al., 2019), provided only a marginal improvement to REM detection, but markedly improved three-stage kappa, due to improved NREM and W classification (kappa score of 0.72 and mean REM F1 score of 0.62).

The ranked importance of features for REM detection, when using all signals, established EOG features as some of the most important (See Fig. 2 (a)). The prevalence of EOG features can be attributed to their correlation with REM sleep and specifically detecting rapid eye movement through permutation entropy, max peak amplitude, and coastline features. In the ranking order, these features are closely followed by elapsed time and EMG features that measure muscle atonia (amplitude percentile, entropy, and relative power). The addition of ECG features did not improve EOG, EMG, or EOG and EMG combinations, indicating that these features are often redundant and perhaps only help to reduce variation (see Fig. 1). While ECG features did not appear fruitful in automated sleep staging, they had some success in RBD detection.

ECG metrics proved effective but ultimately did not outperform EMG metrics in the classification of RBD (see Fig. 3). The metric importance ranking for RBD detection (see Fig. 2 (b)) indicated that NREM origin count and NREM irregular index as the most informative ECG metrics. These metrics are consistent with literature that describe a loss in HRV regulation (Postuma et al., 2010, Sorensen et al., 2013, Bugalho et al., 2018) and differences in atrial fibrillation (Hong et al., 2019) in individuals diagnosed with RBD. Combining EMG and ECG metics provided a marginal improvement in RBD detection, with regards to the F1 score (Fig. 3). This suggests that ECG and EMG metrics are correlated, in that a reduction of HRV regulation during REM is synonymous with a loss of muscle atonia. The impact of automated sleep staging (detailed in Fig. 1) on RBD detection was also evaluated using the most successful combinations of sensors.

The performance of RBD detection using various sensor combinations (shown in Table 2 and Table 3) for both automated sleep staging and RBD classification is detailed in Fig. 4. The boost in performance from using more than one sensor for sleep staging can be observed in C2E1, C2D2, and A3D2 (multiple sensors) compared to B1D1, B1E1, and B1D2 (single EOG sensor). Previously our results confirmed that C2 and A3 provided better REM detection sensitivity compared to B1 (Fig. 1), thereby enabling better RBD detection through better REM classification. Interestingly, when using automated sleep staging (C2 – EOG and EMG), the accuracy of RBD detection achieved was equivalent when using either ECG metrics (C2E1) or EMG metrics (C2D2), see Fig. 4, once again providing evidence that there is considerable overlap in the discriminating abilities of both these metrics, where performance might be differentiated with better REM detection. In terms of achieving economy and efficiency, C2E1 used two sensors (EOG and EMG) compared to three for C2D2 and A3D2 (EOG, EMG and ECG), a potential advantage for a simple and cost-effective screening tool. Furthermore these results are comparable to results derived using EEG features illustrated by the Z3E1 combination, thereby bypassing the complications of applying EEG electrodes.

Bland-Altman analysis was used to evaluate the agreement between metrics using manually and automatically annotated sleep staging from the C2 combination (EOG and EMG). Fig. 5 (a)–(c) provides the B&A plots for the best metrics for RBD detection using an EMG channel (atonia index, fractal exponent, and atonia index ratio). From these plots it is clear that automated sleep staging (derived from EOG and EMG features) sufficiently produced RBD metrics that were correlated to metrics derived from manually annotated sleep staging. However we can observe a bias introduced to RBD metrics derived from automatically annotated sleep staging, where the mean difference (solid black line in Fig. 5) and its confidence interval (grey shaded area) does not include the line of equality (zero axis, dotted line through the origin). This was the direct result of automated sleep stage misclassification. When REM was misclassified as NREM and W, a bias towards values associated with NREM and W sleep was evident. Because automated sleep staging was poorest in RBD participants (red circles), we observed a greater variation in the y-axis (difference), especially as the x-axis values (mean value) moved towards values associated with RBD (red circles). Note that the limits of agreement are calculated by assuming that the difference between the metrics calculated from manually and automatically annotated sleep stages are normally distributed, however this is not necessarily required (Bland and Altman, 1999). From these plots we can observe the resilience and fidelity of these metrics using automated sleep staging from the most successful combination of minimal PSG sensors (EOG and EMG).

These results validate the use of only an EOG and EMG sensor for automated RBD detection, enabling the potential for a minimal, cost-effective, and readily accessible take-home sleep device for RBD screening purposes. However, often epidemiological studies incur much greater costs for logistical reasons and include additional sensors (including ECG and EEG sensors) to optimise their collection of data. A common weakness in existing PSG studies are the inconsistent and intermittent signals that potentially plague recordings. By exploring various sensor combinations, this study also demonstrates the redundancy and ability of PSG sensors to function interchangeably for both automated sleep staging and RBD detection.

## 6 Limitations & future direction

While this study included a large number of participants, its application to a clinical setting is limited because it simply focused on HC and RBD cohorts. A clinical application would demand greater resilience to a myriad of sleeping disorders and other population variations that could easily share confounding RBD attributes. These include periodic limb movement of sleep, OSA, parasomnias, and severe insomnia, which should also be included and evaluated. While ECG metrics did not prove as effective in RBD detection as EMG metrics, there is still no clear indication of which characteristic manifests earlier in RBD participants; loss of REM atonia or loss of HRV regulation during sleep. The loss of HRV regulation might even provide an early bio-marker for RBD, and could potentially offer insight into the phenoconversion of RBD participants, although literature exists that suggests there is currently no correlation for the latter (Postuma et al., 2010). This study also confirmed that successful REM detection is paramount to effective RBD detection and future work to improve automated REM detection would only prove beneficial. Advances in deep learning techniques are yielding promising results and may prove useful in this context (Supratak et al., 2017, Phan et al., 2019). Through the use of limited PSG, this study verifies the feasibility of a simple and practical take-home device for sleep evaluation, of which only a limited number of clinical grade devices supported by literature exist (Mikkelsen et al., 2017, Sterr et al., 2018). Applying such wearable tools would be the next logical step forward towards validating a fully automated RBD detection pipeline.

## 7 Conclusion

This study proved the feasibility of a fully automated pipeline for RBD detection using an EOG and EMG sensor. This study achieved automated sleep staging comparable to manual annotation, which translated to a high performance in RBD detection. Once more this study demonstrated that RBD detection through ECG based metrics was effective but did not out-perform EMG based metrics. Furthermore their use in automated sleep staging provided very little benefit and may not be worth the additional sensor for a minimalistic and economical RBD screening tool.

## Declaration of Competing Interest

The authors declare that they have no known competing financial interests or personal relationships that could have appeared to influence the work reported in this paper.
